# Shallow and reverse attention network for colon polyp segmentation

**DOI:** 10.1038/s41598-023-42436-z

**Published:** 2023-09-14

**Authors:** Go-Eun Lee, Jungchan Cho, Sang-II Choi

**Affiliations:** 1 Department of Computer Science and Engineering, Dankook University, Yongin, 16890 South Korea; 2 School of Computing, Gachon University, Seongnam, 13120 South Korea

**Keywords:** Diseases, Gastroenterology

## Abstract

Polyp segmentation is challenging because the boundary between polyps and mucosa is ambiguous. Several models have considered the use of attention mechanisms to solve this problem. However, these models use only finite information obtained from a single type of attention. We propose a new dual-attention network based on shallow and reverse attention modules for colon polyps segmentation called SRaNet. The shallow attention mechanism removes background noise while emphasizing the locality by focusing on the foreground. In contrast, reverse attention helps distinguish the boundary between polyps and mucous membranes more clearly by focusing on the background. The two attention mechanisms are adaptively fused using a “Softmax Gate”. Combining the two types of attention enables the model to capture complementary foreground and boundary features. Therefore, the proposed model predicts the boundaries of polyps more accurately than other models. We present the results of extensive experiments on polyp benchmarks to show that the proposed method outperforms existing models on both seen and unseen data. Furthermore, the results show that the proposed dual attention module increases the explainability of the model.

## Introduction

Polyps occurring in the colon are divided mainly into benign and malignant types. Even benign polyps, however, involve a risk of developing into colorectal cancer that increases with the size of the polyp, reaching up to 30%^1^. Therefore, early detection and removal of benign polyps through regular colonoscopy can effectively prevent colorectal cancer.

To detect polyps more accurately and effectively using colonoscopy, computer-aided diagnosis (CAD) methods that apply image processing and machine learning techniques to colonoscopy images have been developed. In early studies on CAD techniques^2–5^, handcrafted features were extracted to represent characteristics of polyps such as their color, texture, and shape. Then, the features were classified according to whether they corresponded to polyps with classification models based on machine learning. In the study conducted by Mamonov et al.^2^, the polyp areas in images were detected using geometric analysis and texture-context information. Segmentation was then performed through binary classification of each pixel based on the external features of the polyp. Tajbakhsh et al.^3^ proposed a hybrid context-shape polyp detection method. This approach integrated shape and context information related to the polyp’s appearance in an image. They excluded non-polyp information using context information, and used shape information to predict the location of polyps. Agrahari et al.^6^ utilized an edge detector based on discrete singular convolution to capture the features of a polyp. However, because these methods used only predefined features, their expressive power and classification accuracy were limited.

With the recent rapid development of deep learning-based image analysis technology, various studies^7–12^ have also applied deep learning for medical image analysis. Compared to natural images, medical images contain considerable noise, and the boundaries of objects appearing in such images tend to be ambiguous. Thus, existing general image segmentation techniques cannot be applied straightforwardly to medical images. UNet^13^ is a representative deep learning-based segmentation method for medical images, which consists of contracting and expanding paths into a “U”-shaped structure to effectively segment cell regions in microscopy image data using a skip connection between low- and high-level feature maps. However, because colorectal polyps in endoscopic images exhibit considerable inter-class variation (in color, size, location, and so forth), and boundaries between polyps and mucosa are unclear, the original UNet^13^ cannot learn the characteristics and boundary information of such polyps effectively.

To address this problem, several segmentation models have been developed for polyps in colonoscopy images. SFA^14^ used two decoder structures that shared the same encoder. Each decoder extracted information on the area and boundary of a polyp. They then aggregated the information generated by various kernels using a selective kernel module. Similarly, Psi-Net^15^ was designed to learn three tasks simultaneously by generating contour and distance maps and predicting a mask. In addition to methods^15–17^ for adding branches to extract information about boundaries, models using practical attention modules^8,18–20^ have been developed for natural images. Attention modules improve the performance of polyp detection methods by enabling a model to focus on the visual features of polyps. SANet^19^ used an attention module to remove background information from images to train a model to focus on the foreground. However, SANet needs to fully utilize information about objects’ boundaries. SwinE-Net^21^ connected heterogeneous encoders (Swin Transformer^22^ and EfficientNet model^23^) in parallel and constructed a composite feature map using attention to features extracted from each network. PSNet^24^ used unique dual encoder-decoder structure to improve model’s capabilities. Although these method has the advantage of synergy between heterogeneous networks, it requires relatively heavy network models, with correspondingly large computational resource requirements. PraNet^18^ was designed to predict boundaries more clearly by using reverse attention. However, although various types of information were extracted from multiple layers of the network, these methods, including PraNet^18^, only partially utilized the diversity of information because they use a single type of attention.

We propose a model, “Shallow and Reverse Attention Network” (SRaNet), as a new deep learning model designed to segment polyps more effectively by considering the characteristics of colonoscopy images. We adopted a Res2Net^25^ model using a multi-scale receptive field as a backbone to extract the features of colonoscopy images. To refine the polyp location information in an image and effectively extract detailed boundary information, we also propose a dual attention module called a shallow-reverse dual attention module (SR-DAM).

**Figure 1:**
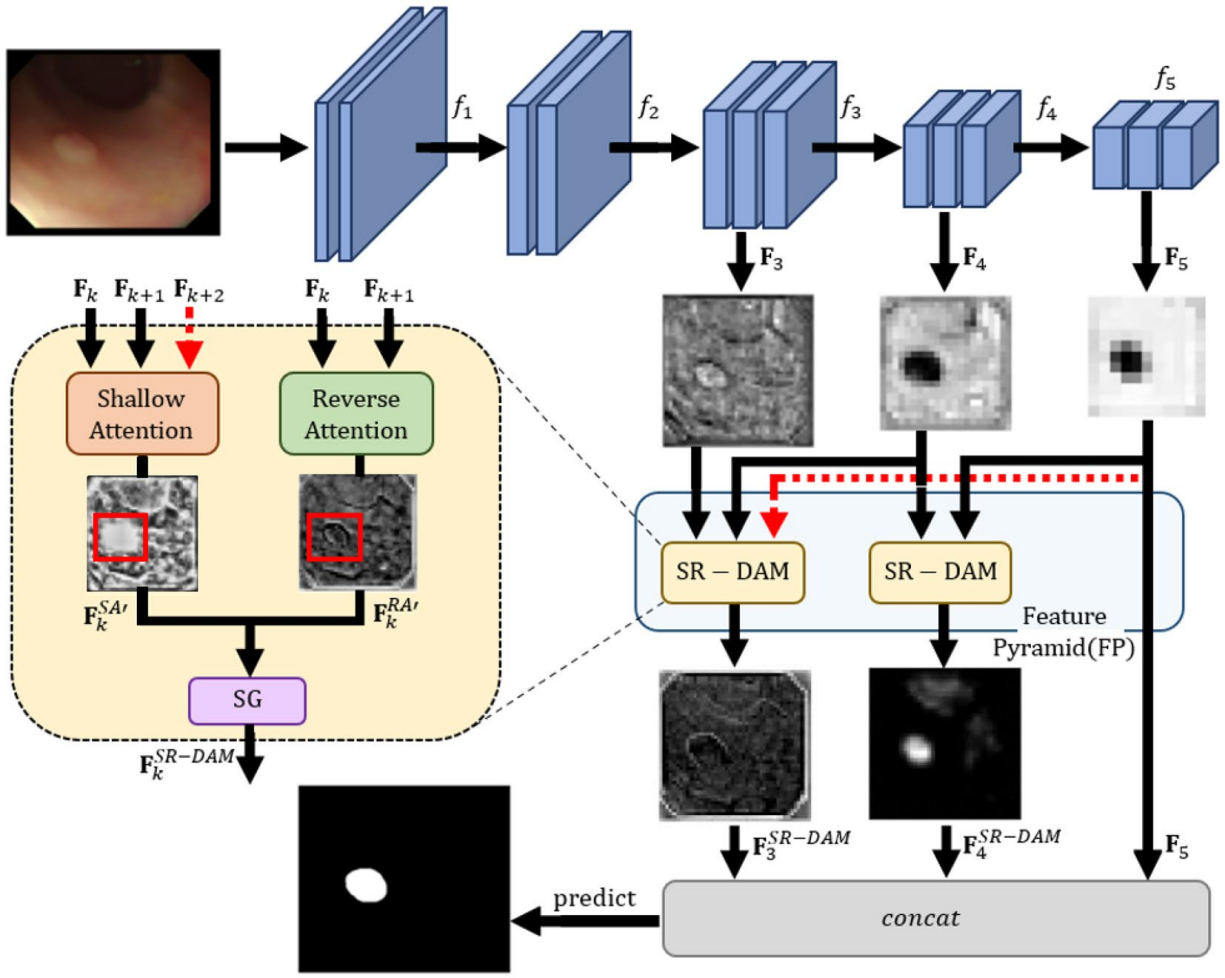
Overview of our proposed network, SRaNet. SRaNet applies shallow-reverse dual attention modules (SR-DAM) to high-level features extracted from Res2Net. Then, using a softmax function, SRaNet fuses different two attention maps adaptively. Our proposed model can learn various information on polyps corresponding to the characteristics of datasets. The red line indicates the flow in case of using more high-level feature maps (${{\textbf {F}}}_{k+2}$). Significantly, the highlighted regions within the red boxes in the attentional results demonstrate that the shallow attention primarily concentrates on the polyp’s foreground. In contrast, the reverse attention specifically targets the polyp’s boundaries.

Figure 1 shows the overall structure of the proposed SRaNet. When the resolution of the feature map falls below a certain level as it passes through the initial layers of the network, SR-DAM is applied between the feature maps in the previous and next layers. Shallow attention removes background information by focusing on high-level and low-level feature maps simultaneously. Thus, we remove background information that acts as noise and maintain contextual information such as the size and shape of the foreground. Conversely, reverse attention applies attention to a reverse map created by performing a reverse operation on the feature map. Reverse attention can be used to accurately classify the boundary between the mucosa and a polyp by learning information about the ambiguous boundary, which has been a challenging issue in polyp data. The proposed SR-DAM can capture correlations between the two feature maps generated by shallow and reverse attention mechanisms. To effectively combine the two kinds of generated attention maps, we designed a “softmax gate (SG).” We apply the softmax function to each channel of the two attention maps to calculate the channel weight between the two attention maps. Then, by multiplying this weight by the existing attention maps and adding them, the proposed approach can differentially utilize the polyp position and attention to the boundary for each channel of the feature map. Consequently, SRaNet effectively extracts refined information about the foreground (polyp) and detailed information about the polyp’s boundary using the above two types of attention in the shared feature map. In addition, the use of attention can increase the explainability of the segmentation result because it enables an intuitive analysis of the mechanisms by which the model detects the object.

The main contributions of this study are summarized as follows.

- We propose a shallow-reverse dual attention module (SR-DAM) that combines shallow attention and reverses attention. These two attentions operate in a complementary manner and improve the explainability of the model.
- The two attentions of SR-DAM are mixed by a softmax gating mechanism in channel dimensions to adaptively determine channel-wise module importance vectors according to the input image.
- The results of extensive experiments demonstrate that the proposed method outperformed other dual attention methods as well as a dedicated polyp segmentation method on benchmark datasets.

## Related works

### Medical image segmentation

Early studies on medical image segmentation extracted handcrafted features to represent characteristics of objects. Image segmentation was performed by training a classifier with the extracted features. However, the performance of these methods was limited because they only used information based on human intuition. As various studies on deep learning and the datasets accessible to researchers have developed over the past years, deep learning-based approaches have been proposed for medical segmentation. Among these, UNet^13^ is widely used in the medical field. UNet consists of an encoder and decoder that respectively extract the features of the object in the image and predict a mask with the information. In addition, UNet can obtain fine-grained details using a skip connection to fuse the feature maps of the corresponding level of the encoder and decoder. Since the introduction of UNet, many works on medical image segmentation have modified the model for improved performance. UNet++^26^ made UNet denser by adding convolution blocks and skip connections between blocks for sophisticated segmentation. UNet3+^27^ captured more fine-grained details by concatenating considerable information on the feature maps of the corresponding layer, as well as feature maps of the different layers of the encoder. These models are commonly used in various medical applications such as brain MRI and X-ray imaging.

Segmentation models^7,14,28,29^ targeting polyp datasets have also been studied and early approaches adopted UNet models. These methods were designed to extract various colors, sizes, and boundary information of polyps in an image. ResUNet++^7^ used atrous spatial pyramid pooling (ASPP) to re-sample multi-scale feature maps with ResUNet^30^, which was proposed for road datasets, to a polyp dataset. It improved the performance of polyp segmentation by adopting the squeeze and excitation (SE)^31^ technique to re-calibrate inter-channel information for a better representation. The method proposed by Jha et al.^28^ added a conditional random field^32^ and test-time augmentation to ResUNet++ for polyp segmentation. SFA^14^ comprised a shared encoder and two decoders designed to predict areas and boundaries. It used a selective kernel module to adaptively extract features from kernels of various sizes. Psi-Net^15^ consisted of three parallel decoder branches to predict mask, contour, and distance maps. The mask branch learned segmentation prediction, and the contour and distance maps were used as auxiliary branches to capture the shape and boundary information of polyps. BDG-Net^33^ obtained a boundary distribution map (BDM) from high-level features using a boundary distribution generation module (BDGM). The map was used as complementary information in a boundary distribution guided decoder (BDGD). Park et al.^34^ employed a teacher-student concept and consistency training to leverage unlabeled data in a semi-supervised manner.

### Image segmentation using attention

With attention mechanisms showing effective performance in natural language processing^35^, several studies have applied attention to natural and medical images. Attention can improve the performance of various computational models by learning the regions of images to focus on to make a final prediction. SENet^31^ used a “squeeze and excitation” block to extract and recalibrate important information between channels by utilizing channel attention. Attention UNet^36^ was developed for pancreatic segmentation in abdominal CT. Adding an attention gate to UNet refined the encoder information and passed the information to the skip connection. For colonoscopy images, PraNet^18^ was designed to focus on ambiguous boundaries between colon polyps and mucosa by paying attention to reversed feature maps. SANet^19^ extracted complementary information by focusing on high- and low-level feature maps. CaraNet^20^ detected small colorectal polyps by adding an axial attention mechanism to PraNet’s reverse attention. MCDALNet^37^ used dual attention to utilize position and channel information. Recently, some models using transformer with multi-head self-attention (MHSA) have been studied^10,29,38,39^. Polyp-PVT^10^ and ColonFormer^29^ used a modified transformer for medical segmentation tasks. Although many works have explored the use of attention to improve the performance of polyp segmentation methods, we focused on the attention mechanisms of SANet^19^ and PraNet^18^, which are relatively intuitive and can effectively utilize polyp boundary information.

## Methods

As depicted in Fig. 1, the proposed method consists of two types of attention, including shallow and reverse attention, to capture different characteristics of feature maps extracted from a Res2Net backbone^25^. Dual attention is performed using three high-level feature maps of the feature pyramid (FP) structure. We utilized these feature maps to extract detailed semantic information for polyp detection. After dual attention, a softmax gating mechanism is used to combine the two attention feature maps based on their importance.

### Preliminary

Attention is a technique used to learn which parts of feature maps are more important than others. Owing to its effectiveness, several studies have also considered modeling contextual information using multiple complementary types of attention. CBAM^40^ sequentially inferred channel and spatial attention based on an input feature map and then multiplied attention maps to the feature map for adaptive feature refinement. DANet^41^ applied a self-attention mechanism to channel and position dimensions. The two self-attention modules enhanced interdependent information along channel and location dimensions, and their output and two feature maps were merged via sum fusion to further improve the representation of features. MANet^42^ addressed the under-utilization of multi-scale features in U-Net^13^ by extracting contextual dependencies through both kernel and channel attention. MCDALNet^37^ integrated channel-position dual attention modules into a U-Net-type decoder to capture the channel and spatial dependencies of feature maps to help improve the discrimination of features in medical image segmentation.

However, as demonstrated in our experiments, there is scope of improvement in terms of the performance of these methods for medical images. In medical images, the shapes of targets are typically inconsistent, and their boundaries are ambiguous. Therefore, focusing on both boundaries and spatial locations is important. In this study, we propose a dual attention-based method that combines complementary shallow attention and reverse attention mechanisms designed to extract contextual information from boundaries. In addition, the existing channel-position attention modules are typically difficult to interpret when visualizing the parts of a feature map on which the model focused. However, the proposed method can intuitively visualize the inference process for better explainability.

### Shallow and reverse attention

In general, the more feature maps obtained at the deep level of the network, the larger is the receptive field, which makes the background of the feature map cleaner. However, this also renders the boundaries more ambiguous. Shallow attention uses a shallow (low-level) feature map with local information, and a deep (high-level) feature map with object information together. By combining foreground information from the deep feature map with local information from the shallow feature map, shallow attention removes background noise features effectively and simultaneously emphasizes local features. To obtain a refined feature map in which the noisy background has been removed, we multiply the shallow and deep feature maps.

**Figure 2:**
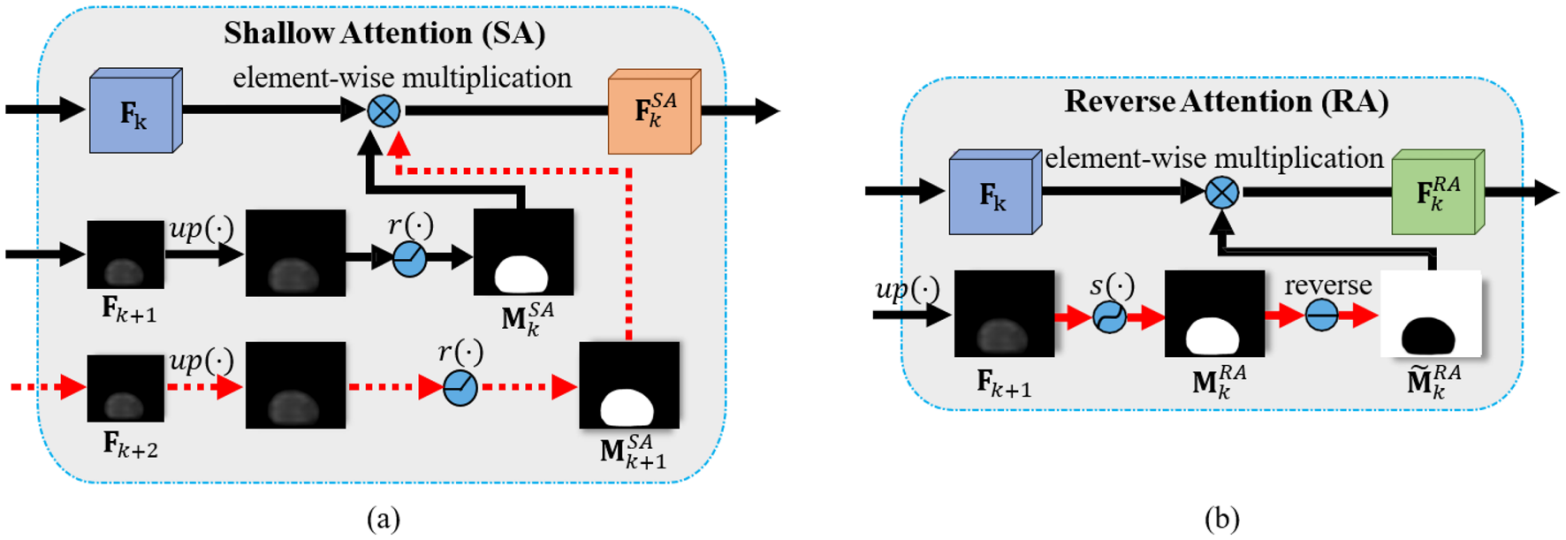
(**a**) Shallow attention module and (**b**) reverse attention module.

The shallow attention module (SAM) is shown in Fig. 2a. Let ${{\textbf {F}}}_{k} \in {\mathbb {R}}^{L\times M\times N}$ be the *k*-th feature map in Fig.  1, where *L*, *M*, and *N* are respectively the numbers of rows, columns, and channel dimensions. If ${{\textbf {F}}}_{k}$ has only one high-level feature map, as shown in Fig. 2a (marked by black arrows), shallow attention map ${{\textbf {M}}}_{k}^{SA}$ and ${{\textbf {F}}}_{k}^{SA}$ of the SAM can be represented as

1 $$\begin{aligned} {{\textbf {M}}}_{k}^{SA} = r\left( up\left( {{\textbf {F}}}_{k+1}\right) \right) ,\quad \quad {{\textbf {F}}}_{k}^{SA} = {{\textbf {M}}}_{k}^{SA}\otimes {{\textbf {F}}}_{k} \end{aligned}$$

where $r(\cdot )$ is a ReLu activation function and $\otimes$ is element-wise multiplication. Here, the upsampling function $up(\cdot )$ is used to match the resolution of the deep-level feature map ${{\textbf {F}}}_{k+1}$ to the shallow-level resolution ${{\textbf {F}}}_{k}$. If ${{\textbf {F}}}_{k+2}$ exists, which is a layer higher than ${{\textbf {F}}}_{k+1}$ in the *k*-th layer, we also use ${{\textbf {F}}}_{k+2}$ for shallow attention as higher-level (context) information and obtain ${{\textbf {M}}}_{k+1}^{SA}$ as follows (Fig. 2a (marked by red arrows)).

2 $$\begin{aligned} {{\textbf {M}}}_{k+1}^{SA} = r\left( up\left( {{\textbf {F}}}_{k+2}\right) \right) , \quad \quad {{\textbf {F}}}_{k}^{SA} = {{\textbf {M}}}_{k}^{SA}\otimes {{{\textbf {M}}}_{k+1}^{SA}} \otimes {{\textbf {F}}}_{k} \end{aligned}$$

Reverse attention helps distinguish the boundary between the polyp and mucosa more clearly. Figure 2b shows the reverse attention module (RAM). As in Fig. 2b, a normalized attention map ${{{\textbf {M}}}}_{k}^{RA}$ is first created by applying a sigmoid activation function and an upsampling operation to the higher-level feature map ${{\textbf {F}}}_{k+1}$. Here, the sigmoid function is used to normalize the feature values from 0 to 1. Subsequently, a reverse attention map $\tilde{{{\textbf {M}}}}_{k}^{RA}$ containing information about the background is generated by reversing ${{{\textbf {M}}}}_{k}^{RA}$. Multiplying this attention map with the previous level’s feature map ${{\textbf {F}}}_k$ for the foreground enables the model to focus on the object’s boundary. The attended feature map ${{\textbf {F}}}_{k}^{RA}$ for ${{\textbf {F}}}_k$ is obtained as follows.

3 $$\begin{aligned} {{{\textbf {M}}}}_{k}^{RA} = s\left( up\left( {{\textbf {F}}}_{k+1}\right) \right) , \quad \quad \tilde{{{\textbf {M}}}}_{k}^{RA} = {{\textbf {1}}} - {{{\textbf {M}}}}_{k}^{RA}, \quad \quad {{\textbf {F}}}_{k}^{RA} = \tilde{{{\textbf {M}}}}_{k}^{RA}\otimes {{\textbf {F}}}_{k} \end{aligned}$$

Notably, RAM emphasizes background noise on ${{\textbf {F}}}_{k}$, whereas the ground truth does not include background noise. Thus, by training the model to reduce this difference, feature maps with clean backgrounds can be generated. Accordingly, unlike SAM, RAM may further emphasize the boundary between polyps and mucous membranes.

The above two attention (shallow and reverse) modules in the proposed dual-attention structure (SR-DAM) are complementary to each other; SAM emphasizes the local features of the foreground, and RAM has characteristics that emphasize boundary information, including background information. By mixing these two attention modules, the model can predict an accurate boundary for the foreground and reduce the noise in the background as well.

### Softmax gate

**Figure 3:**
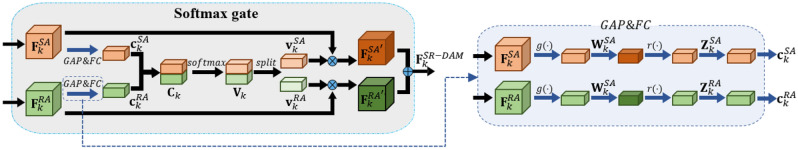
Softmax gate (SG). We fuse two feature maps extracted from SR-DAM using softmax gate by their importance. Therefore, our proposed model can learn information of polyps according to the characteristic of different datasets.

Depending on the type of polyp dataset, the characteristics on which to focus during segmentation may differ. For datasets with noisy backgrounds, information from SAM is important, whereas for datasets with ambiguous boundaries, information from RAM is important. To handle unknown data effectively, we propose a “softmax gate (SG)”, which aggregates the two attention maps by performing channel reweighting as shown in Fig.  3.

To effectively utilize the results of SAM and RAM, we perform global averaging pooling $g(\cdot )$ followed by two fully connected layers (*FC*) and a ReLu function $r(\cdot )$, respectively, as in SENet^31^. Channel importance vectors (${{\textbf {c}}}_{k}^{SA}\in {\mathbb {R}}^{1 \times N}$ and ${{\textbf {c}}}_{k}^{RA}\in {\mathbb {R}}^{1 \times N}$, where *N* is the number of channels), which indicate the importance of ${{\textbf {F}}}_{k}^{SA}$ and ${{\textbf {F}}}_{k}^{RA}$ by channel, are obtained as follows.

4 $$\begin{aligned} {{\textbf {c}}}^{\{SA,RA\}}_{k} = {{\textbf {Z}}}^{\{SA,RA\}}_{k}\cdot r\left( {{{\textbf {W}}}}^{\{SA,RA\}}_{k}\cdot g\left( {{\textbf {F}}}_{k}^{\{SA,RA\}}\right) \right) \end{aligned}$$

where ${{{\textbf {W}}}}^{\{SA,RA\}}_{k}, {{\textbf {Z}}}^{\{SA,RA\}}_{k}$ are the weights of the fully connected layer of the importance vectors. Here, global averaging pooling (GAP) abstracts the global spatial information into a channel descriptor and calculates the channel-wise dependency by recalibrating the abstracted information through two fully connected layers (${{{\textbf {W}}}}^{\{SA,RA\}}_{k}$ and ${{\textbf {Z}}}^{\{SA,RA\}}_{k}$).

We concatenate ${{\textbf {c}}}^{SA}_{k} \in {\mathbb {R}}^{1\times N}$ and ${{\textbf {c}}}^{RA}_{k} \in {\mathbb {R}}^{1\times N}$ along the channel direction to yield ${{\textbf {C}}}_{k} = \left[ {{\textbf {c}}}^{SA}_{k}; {{\textbf {c}}}^{RA}_{k}\right] \in {\mathbb {R}}^{2\times N}$, and apply a softmax function to each channel, resulting in ${{\textbf {V}}}_{k} = softmax({{\textbf {C}}}_{k})$. Subsequently, we divide ${{\textbf {V}}}_{k}$ into modules, yielding ${{\textbf {V}}}_{k} =\left[ {{\textbf {v}}}^{SA}_{k}; {{\textbf {v}}}^{RA}_{k}\right]$. From this, we derive two channel-wise module importance vectors, ${{\textbf {v}}}^{SA}_{k}$ and ${{\textbf {v}}}^{RA}_{k}$. By multiplying ${{\textbf {v}}}^{SA}_{k}$ and ${{\textbf {v}}}^{RA}_{k}$ element-wise with each channel of ${{\textbf {F}}}_{k}^{SA}$ and ${{\textbf {F}}}_{k}^{RA}$, we generate ${{{\textbf {F}}}_{k}^{SA}}^{\prime }$ and ${{{\textbf {F}}}_{k}^{RA}}^{\prime }$. Each element of these outputs is readjusted by SAM and RAM, respectively. This softmax normalization process helps mitigate internal covariance shift during training. Furthermore, it transforms the distribution of the elongated loss function into a spherical one, thereby enhancing the model’s learning effectiveness. Finally, ${{{\textbf {F}}}_{k}^{SA}}^{\prime }$ and ${{{\textbf {F}}}_{k}^{RA}}^{\prime }$ are combined to produce the aggregated feature map ${{\textbf {F}}}^{SR-DAM}k$, such that ${{{\textbf {F}}}^{SR-DAM}k = {{\textbf {F}}}_{k}^{SA}}^{\prime } + {{{\textbf {F}}}_{k}^{RA}}^{\prime }$. As highlighted in the method presented by Wu et al.’s method^43^, using low-level feature maps demands more computational resources than high-level features due to their resolution. Therefore, we applied our SR-DAM to three high-level feature maps, as illustrated in Fig. 1.

The proposed model is trained by using the following loss function:

5 $$\begin{aligned} {{\textbf {L}}}(y, {\hat{y}})_{DICE} = 1 - \frac{2 \cdot \sum {y \cdot {\hat{y}}}}{\sum {y^2} + \sum {{\hat{y}}^2} + \varepsilon }, \quad {{\textbf {L}}}_{BCE}(y, {\hat{y}}) = - \left( y \cdot \log ({\hat{y}}) + (1 - y) \cdot \log \left( 1 - {\hat{y}}^2\right) \right) , \quad {{\textbf {L}}}_{total} = {{\textbf {L}}}_{DICE} + {{\textbf {L}}}_{BCE} \end{aligned}$$

where ${{\textbf {L}}}_{DICE}$ and ${{\textbf {L}}}_{BCE}$ represent the weighted dice score and binary cross entropy (BCE) loss between the ground-truth y and prediction label ${\hat{y}}$, respectively.

## Results

### Experimental setup

***Implementation details*** For fairness, we used the same backbone, Res2Net^25^ for PraNet^18^ and SANet^19^. The number of training epochs and the batch size were set to 128 and 64, respectively. The initial learning rate was set to 0.004 and 0.4 for the feature extraction backbone and the segmentation head, respectively. The learning rate decreased by a factor of 0.5 every 32 epochs. The proposed model was trained on multi-scale images by resizing the training image to $352\times 352$ and randomly cropping among [256, 288, 320, 352] with probabilities of [0.1, 0.2, 0.3, 0.4], respectively. For data augmentation, random flips (horizontal and vertical) and 90-degree rotations were randomly applied with a probability of 50%. We adopted color exchange as in SANet^19^. In addition, to compare with the latest transformer-based polyp segmentation methodologies, we performed experiments where the backbone of our proposed method was switched to a pyramid vision transformer (PVT)^44^. We conducted our experiments on a workstation powered by an Intel Xeon W-2245 CPU, a GeForce RTX 3090 GPU, and 32GB DDR4 RAM using PyTorch framework. We repeated experiments ten times to evaluate our method and reported the average values and standard deviations to enhance statistical reliability. When using Res2Net and PVT as backbone respectively, the proposed method achieves a real-time processing speed of about 50 and 45 frames per second for $352\times 352$ inputs.

**Table 1:** Quantitative comparison of our proposed SRaNet on polyp benchmarks Kvasir and ClinicDB.

	Kvasir						ClinicDB					
	mDice	mIoU	$F_{\beta }^{\omega }$	$S_{\alpha }$	$E_{\xi }^{max}$	MAE	mDice	mIoU	$F_{\beta }^{\omega }$	$S_{\alpha }$	$E_{\xi }^{max}$	MAE
UNet	0.818	0.746	0.794	0.858	0.893	0.055	0.823	0.755	0.811	0.889	0.954	0.019
UNet++	0.821	0.743	0.808	0.862	0.910	0.048	0.794	0.729	0.785	0.873	0.931	0.022
ResUNet++	0.813	0.793	na	na	na	na	0.796	0.796	na	na	na	na
PraNet	0.898	0.840	0.885	0.915	0.948	0.030	0.899	0.849	0.896	0.936	0.979	0.009
SANet	0.904	0.847	0.892	0.915	0.953	0.028	0.916	0.859	0.909	0.939	0.976	0.012
TransUNet	0.913	0.857	na	na	na	na	0.935	0.887	na	na	na	na
Polyp-PVT	0.917	0.864	0.911	0.925	0.962	0.023	**0.937**	**0.889**	**0.936**	0.949	**0.989**	**0.006**
CBAM	0.823	0.738	0.812	0.867	0.913	0.042	0.890	0.831	0.890	0.931	0.976	0.011
DANet	0.892	0.833	0.881	0.911	0.943	0.033	0.912	0.851	0.903	0.936	0.970	0.015
**Proposed**	0.910	0.857	0.898	0.912	0.922	0.025	0.911	0.858	0.908	0.942	0.975	0.013
Proposed*	**0.921**	**0.870**	**0.921**	**0.932**	**0.970**	**0.019**	0.926	0.875	0.933	**0.950**	0.983	**0.006**

The results of the dual attention-based approach (CBAM and DANet) are shown in their performance on polyp datasets. Among these, CBAM was modified for the segmentation task.

* means that we used a pyramid vision transformer as a backbone network. The best results are in bold.

***Datasets*** We evaluated the proposed method using the same experimental settings as in PraNet^18^. Five benchmark datasets were used for the evaluation: CVC-ClinicDB^45^, Kvasir^46,47^, CVC-300^18,48^, CVC-ColonDB^3^, and ETIS^49^. Particularly, Kvasir and CVC-ClinicDB were used as training and testing data (seen data). The remaining three datasets were used as testing data to verify the generalization of the model with unseen data.

**Table 2:** Quantitative comparison of our proposed SRaNet on polyp benchmarks ColonDB and ETIS.

	ColonDB						ETIS					
	mDice	mIoU	$F_{\beta }^{\omega }$	$S_{\alpha }$	$E_{\xi }^{max}$	MAE	mDice	mIoU	$F_{\beta }^{\omega }$	$S_{\alpha }$	$E_{\xi }^{max}$	MAE
UNet	0.512	0.444	0.498	0.712	0.776	0.061	0.398	0.335	0.366	0.684	0.740	0.036
UNet++	0.486	0.410	0.467	0.691	0.760	0.064	0.401	0.344	0.390	0.683	0.776	0.035
PraNet	0.712	0.640	0.699	0.820	0.872	0.043	0.628	0.567	0.600	0.794	0.841	0.031
SANet	0.753	0.670	0.726	0.837	0.878	0.043	0.750	0.654	0.685	0.849	0.897	0.015
TransUNet	0.781	0.699	na	na	na	na	0.731	0.660	na	na	na	na
Polyp-PVT	0.808	0.727	0.795	0.865	0.919	0.031	0.787	0.706	0.750	0.871	**0.910**	**0.013**
CBAM	0.608	0.512	0.688	0.803	0.858	0.051	0.399	0.319	0.366	0.679	0.745	0.031
DANet	0.717	0.640	0.700	0.825	0.883	0.040	0.628	0.551	0.612	0.801	0.843	0.022
**Proposed**	0.777	0.700	0.740	0.837	0.890	0.037	0.771	0.688	0.720	0.873	0.890	0.031
Proposed*	**0.814**	**0.734**	**0.804**	**0.877**	**0.927**	**0.026**	**0.788**	**0.707**	**0.751**	**0.897**	0.904	0.016

* means that we used a pyramid vision transformer as a backbone network. The best results are in bold.

**Table 3:** Quantitative comparison of our proposed SRaNet on polyp benchmark CVC-300.

	CVC-300					
	mDice	mIoU	$F_{\beta }^{\omega }$	$S_{\alpha }$	$E_{\xi }^{max}$	MAE
UNet	0.710	0.627	0.684	0.843	0.875	0.022
UNet++	0.707	0.624	0.687	0.839	0.898	0.018
PraNet	0.871	0.797	0.843	0.925	0.972	0.010
SANet	0.888	0.815	0.859	0.928	0.972	0.008
TransUNet	0.893	0.824	na	na	na	na
Polyp-PVT	**0.900**	**0.833**	**0.884**	**0.935**	**0.981**	0.007
CBAM	0.729	0.615	0.693	0.855	0.899	0.019
DANet	0.874	0.796	0.845	0.930	0.975	0.009
**Proposed**	0.899	0.830	0.858	0.911	0.958	**0.006**
Proposed*	0.890	0.823	0.872	0.933	0.978	0.008

* means that we used a pyramid vision transformer as a backbone network. The best results are in bold.

**Table 4:** Comparison of standard deviation (SD) of the mean Dice on polyp benchmarks.

	Kvasir	ClinicDB	ColonDB	ETIS	CVC-300
	mDice±SD	mDice±SD	mDice±SD	mDice±SD	mDice±SD
CBAM	$0.823 \pm 0.051$	$0.890 \pm 0.047$	$0.608\pm 0.042$	$0.399\pm 0.050$	$0.729\pm 0.042$
DANet	$0.892\pm 0.046$	$0.912\pm 0.042$	$0.717\pm 0.038$	$0.628\pm 0.037$	$0.874\pm 0.049$
**Proposed**	$0.910\pm 0.037$	$0.911\pm 0.044$	$0.777\pm 0.033$	$0.771\pm 0.034$	${\textbf {0.899}}\pm 0.048$
Proposed*	${\textbf {0.921}}\pm 0.035$	${\textbf {0.926}}\pm 0.042$	${\textbf {0.814}}\pm 0.034$	${\textbf {0.788}}\pm 0.035$	$0.890\pm 0.040$

The best results are in bold.

***Comparison methods*** Both Dice and intersection over union (IoU) are commonly used to measure the performance of segmentation models by calculating their accuracy predicted among all pixels. We used two metrics for the performance comparison: mIoU (mean IoU) and mDice (mean Dice). To demonstrate the advantages of our proposed method, we’ve also utilized four additional metrics: Weighted F-measure $\left( F_{\beta }^{\omega }\right)$, S-measure ($S_{\alpha }$)^50^, E-measure $\left( E_{\xi }\right)$^51^, and Mean Absolute Error (MAE). The $F_{\beta }^{\omega }$ is an accuracy indicator, computing the harmonic mean of precision and recall. The $S_{\alpha }$ measures structural similarity between predictions and ground truths at the object level. The $E_{\xi }$ is an enhanced-alignment metric utilized to evaluate segmentation results. Lastly, the MAE is a pixel-by-pixel metric that computes the average absolute error between predictions and ground truths.

We compared with five medical image segmentation methods, including UNet, UNet++, ResUNet++, PraNet, and SANet, and two transformer-based methods, TransUNet^39^ and Polyp-PVT^10^. Furthermore, we evaluated the performance of the proposed SR-DAM in polyp segmentation in comparison with that of other dual attention-based methods such as CBAM and DANet. To utilize CBAM for image segmentation, ResNet^30^ was used as the feature extraction backbone and the classification branch was replaced with a segmentation branch. In Tables 1 to  3, we referenced the results for methods other than CBAM, DANet, Proposed, and Proposed*, using values reported in previous studies on polyp segmentation^10^. We assessed the performance of CBAM, DANet, and our model under consistent conditions, including the composition of training and test datasets.

### Quantitative comparison

Tables 1, 2, and 3 show that UNet and its variant models did not perform as well as expected in polyp segmentation, failing to effectively capture the ambiguous boundary characteristics across all datasets. Because SANet and PraNet are designed for medical image segmentation, the attention modules of each model efficiently extracted information about the foreground or background; these methods exhibited improved performance compared to previous models. Our proposed model demonstrated noticeable performance improvements with mean Dice improvements from 1 to 2% and 6 to 15% when compared with SANet and PraNet, respectively, on unseen datasets. Notably, results from our method using a transformer backbone showed significant increases across all metrics. Considering that the proposed model uses the same attention modules, SAM of SANet and RAM of PraNet, the proposed dual attention module aggregates the complementary information from the two attention modules, allowing the model to learn the polyp and its boundary features.

When comparing the two methods DANet and CBAM using dual attention, DANet achieved higher performance than CBAM. The main difference between the two methods is that CBAM aggregates two attention maps sequentially using element-wise multiplication, whereas DANet aggregates them in parallel using self-attention. This indicates that aggregate attention maps in parallel with self-attention is more effective. Table 1 also demonstrates that the proposed method significantly improved the mean Dice score by 2–9% compared to CBAM on seen datasets. As highlighted in Tables 2 and 3, this difference escalates to 17–37% on unseen datasets. Notably, the proposed method achieved substantial performance enhancement compared to DANet, which, like SRaNet, aggregates two attention maps in parallel. This difference becomes more pronounced in the ETIS dataset, known for its small targets. This means that focusing only the attention needed to accurately predict polyps is important, as opposed to the channel-position dual attention commonly used in other methods.

Comparing the performance on each testing dataset, Table 1 shows that SANet and DANet achieved similar performance as our model for the seen datasets (i.e*.*, ClinicDB and Kvasir). However, for the unseen datasets, our model showed the best performance, indicating that our model exhibited better generalization.

While our proposed method utilizing Res2Net as a backbone exhibited a slightly lower performance than the latest transformer-based methods, such as TransUNet^39^ and Polyp-PVT^44^, a notable improvement was observed when we switched the backbone to pyramid vision transformer (PVT)^44^. With this alteration, the performance of our proposed method across various datasets proved to be either superior to or on par with the most recent transformer-based methods. This indicates that the performance enhancement brought about by our proposed module is independent of the specific backbone used.

Table 4 also presents the mean dice coefficient’s standard deviation (SD), comparing our proposed method with other dual attention-based approaches. As demonstrated, our method exhibits lower standard deviations than the others. This implies that our method offers superior stability across all datasets.

***Generalization capability*** We carried out three experiments to evaluate the model’s generalizability across different datasets. Similar to PraNet^18^, the CVC-ColonDB is a collection of 380 images from 15 short colonoscopy sequences. The ETIS dataset comprises 196 polyp images aimed at the early detection of colorectal cancer. Meanwhile, CVC-300 features 300 white-light images extracted from 13 sequences, part of the EndoScene dataset^48^. While each dataset poses unique challenges, SRaNet outperforms traditional medical segmentation methods like U-Net, U-Net++, and attention-based models regarding generalization.

### Qualitative comparison

**Figure 4:**
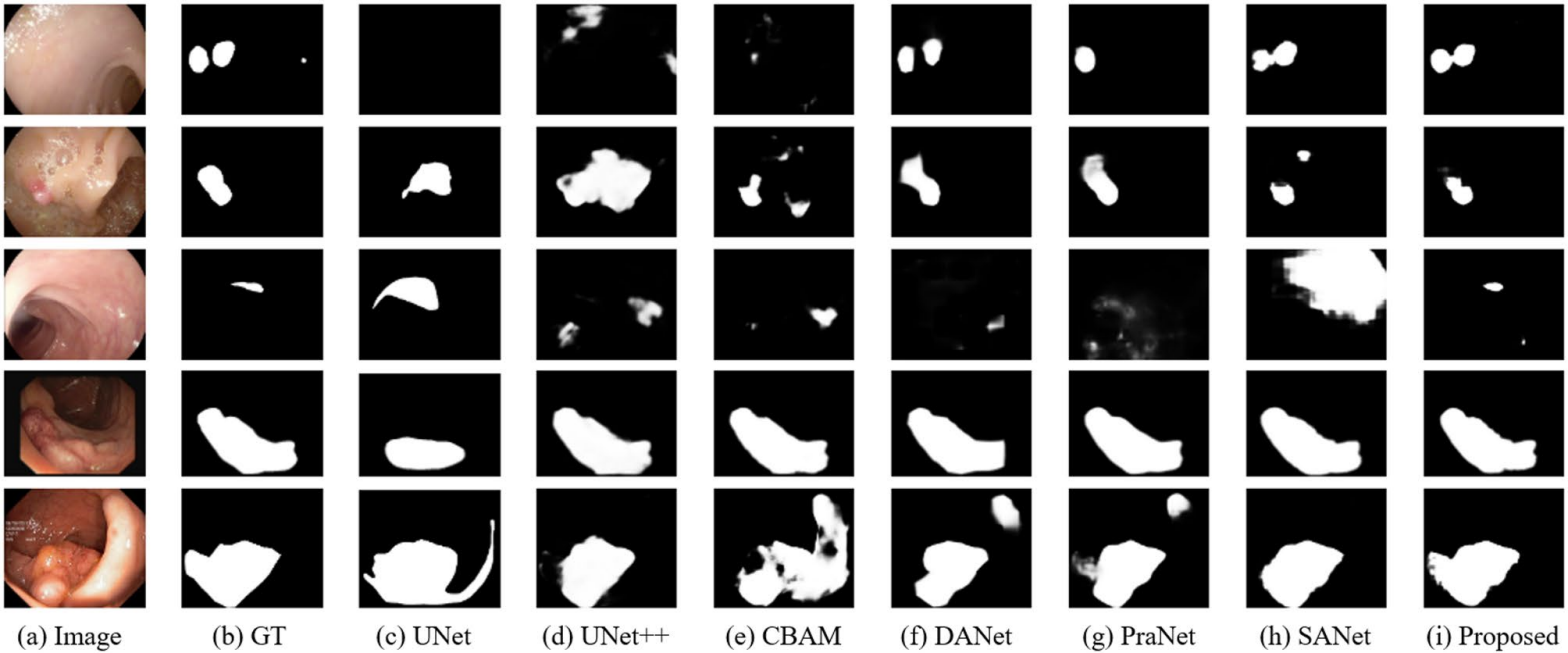
Qualitative comparison. We visualize the prediction mask of medical segmentation SOTA models and dual attention-based models. Images were extracted from each of the five datasets. These results show that our proposed method can detect the location of polyps and the boundary between polyp and mucosa accurately on unseen data as well as seen data.

Figure 4 shows the prediction results of all methods. It may be observed that UNet and UNet++ did not accurately segment the polyp location or its boundary. In addition, CBAM and DANet using dual attention also failed to segment a polyp’s location and boundary accurately. In the case of SANet and PraNet using polyp-dedicated attention, the location of polyps was predicted relatively better than with previous methods. However, the proposed method yields the best results in terms of both locations and boundaries. Particularly, the proposed method predicted cleaner and more accurate boundaries than SANet. In addition, the proposed method accurately recognized the background misclassified by PraNet using RAM. The proposed method used SAM and RAM together to collaboratively supplement the information needed by each module, unlike SANet and PraNet. Consequently, the model achieved more accurate predictions.

### Explainability of dual attention

**Figure 5:**
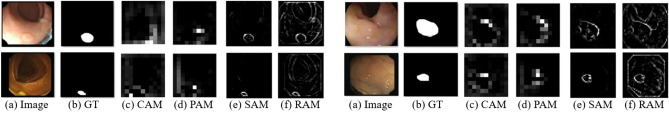
Explainability of shallow and reverse attention. We visualized the feature maps extracted from CAM and PAM of DANet, SAM, and RAM of our proposed method. As shown above, CAM and PAM lack the explanatory power to show how the models learned from images. In contrast, SAM and RAM provide intuitive information about polyps.

Existing dual attention-based models learn objects using channel-position attention, and have demonstrated notable improvements in performance on general image segmentation tasks. However, medical objects, particularly polyps, differ considerably from general objects, and the performance of existing methods is unsatisfactory. Moreover, in the case of medical imaging, the explainability and interpretability of computational models are very important owing to the criticality of the application. Therefore, we compared the explainability of the proposed shallow-reverse dual attention module with existing methods by visualizing the attention map shown in Fig.  5.

Figure 5c and d show feature maps emphasized by the channel-position attention. When analyzing a visualized image, it could be more intuitive to determine the characteristics of the model captured by the image polyp. However, in Fig. 5e and f, visualized using the proposed shallow-reverse attention, the part of the image the model focused on may be easily confirmed. Therefore, the proposed method can increase the explainability of the model in terms of interpreting how it is trained through highly intuitive visual results.

### Ablation study

We conducted an ablation study to analyze the contribution to the performance improvement of each module of the proposed method. In Table 5, “Baseline” represents a baseline model that used only a backbone without any attention. RAM and SAM indicate the attention modules used in the proposed dual attention approach. Thus, the second and third rows represent attention-based models with shallow or reverse attention, respectively, with the same backbone. The fourth row shows the result of a variant model that used shallow attention and reverse attention but with a simple sum aggregation of the two feature maps without a softmax gate. “Sig” represents the use of the sigmoid function when aggregating two attention maps, and “Soft” represents the use of softmax function. Furthermore, we show how leveraging low-level feature maps with detailed information affected the performance of the model in Table 6.

**Table 5:** Effects of our proposed methods.

Baseline	SAM	RAM	Sig	Soft	Kvasir		ClinicDB		CVC-300		ColonDB		ETIS	
					mDice	mIoU	mDice	mIoU	mDice	mIoU	mDice	mIoU	mDice	mIoU
$\checkmark$					0.890	0.826	0.898	0.833	0.885	0.806	0.746	0.663	0.746	0.654
$\checkmark$	$\checkmark$				0.904	0.847	**0.916**	**0.859**	0.888	0.815	0.753	0.670	0.750	0.654
$\checkmark$		$\checkmark$			0.887	0.836	0.889	0.833	0.874	0.807	0.712	0.637	0.645	0.574
$\checkmark$	$\checkmark$	$\checkmark$			0.896	0.836	0.902	0.845	0.888	0.819	0.760	0.674	0.762	0.679
$\checkmark$	$\checkmark$	$\checkmark$	$\checkmark$		0.897	0.839	0.909	0.855	0.893	0.823	0.758	0.677	0.753	0.667
$\checkmark$	$\checkmark$	$\checkmark$		$\checkmark$	**0.910**	**0.857**	0.911	0.858	**0.899**	**0.830**	**0.777**	**0.700**	**0.771**	**0.688**

We show the effectiveness of dual attention module and softmax gating for polyp dataset. “Baseline” means a model using only backbone. “SAM” and “RAM” are attention modules, as illustrated in Fig. 2a and  b, respectively. “Sig” and “Soft” indicate which activation function was used when combining two feature maps extracted from SR-DAM. The best results are in bold.

**Table 6:** Effects of feature pyramid. ${{\textbf {F}}}_{3}$, ${{\textbf {F}}}_{4}$, and ${{\textbf {F}}}_{5}$ refer to the feature maps extracted in Fig. 1.

	Kvasir		ClinicDB		CVC-300		ColonDB		ETIS	
	mDice	mIoU	mDice	mIoU	mDice	mIoU	mDice	mIoU	mDice	mIoU
Baseline	0.890	0.826	0.898	0.833	0.885	0.806	0.746	0.663	0.746	0.654
Baseline (PVT)	0.910	0.857	0.911	0.850	0.890	0.820	0.793	0.707	0.779	0.682
Proposed w/o FP	0.907	0.847	0.899	0.837	0.885	0.810	0.752	0.667	0.744	0.647
Proposed	0.910	0.857	0.911	0.858	**0.899**	**0.830**	0.777	0.700	0.771	0.688
Proposed*	**0.921**	**0.870**	**0.926**	**0.875**	0.890	0.823	**0.814**	**0.734**	**0.788**	**0.707**

Each row represents which feature map the model used to generate the final mask and its result.

* means that we used a pyramid vision transformer as a backbone network. The best results are in bold.

**Table 7:** Computation comparison.

	GMACs	Params(M)
PraNet	13.11	32.55
SANet	11.27	23.80
CBAM	10.21	28.07
DANet	27.00	49.48
Polyp-PVT	10.04	25.11
Proposed	11.28	23.90
Proposed*	9.68	24.91

We calculated the computational complexity and the number of parameters of models using the attention to verify the efficiency of our proposed model.

* means that we used a pyramid vision transformer as a backbone network.

***Dual attention*** The results of the ablation study for dual attention with simple sum aggregation are shown in the first to fourth rows of Table 5. The model using both SAM and RAM (fourth row) achieved lower performance than that using only SAM (second row) on ClinicDB and Kvasir datasets. However, except for the two datasets, which were the seen datasets, dual attention showed a slight performance improvement on the remaining unseen datasets. Notably, the performance improvement was more prominent for the ETIS dataset with tiny polyps and ambiguous boundaries than for the other datasets. This is because the different types of information complemented each other by applying each attention to the image. From this result, we can conclude that the generalization performance was enhanced even through a simple sum of the two attention mechanisms.

***Softmax gate*** The fourth to last rows of Table 5 show variations in performance with the aggregation of the two attention maps. Comparing the performance when using the sigmoid (fifth row) with the simple sum, the overall sigmoid aggregation performance, except for the ETIS dataset, was higher than the simple sum (fourth row). This implies that the attention module operated in accordance with the input image. When using the softmax function, we confirmed that the channel-wise module importance vectors were calculated adaptively and the effect of normalization was also applied, in contrast to a sigmoid, which improved performance on all datasets. In particular, the effectiveness of our proposed Softmax gate can be highlighted by comparing the results from the fourth and sixth rows in Table 5, which utilizes a simple summation method. In the fourth row, SAM and RAM may interfere with each other due to their opposing information learning patterns, potentially acting as noise. On the other hand, the sixth row, featuring the Softmax gate of our proposed method, successfully delineates the channel-level importance between the two modules in a complementary fashion, which subsequently contributes to the overall performance enhancement.

***Feature pyramid*** We analyzed the effectiveness of feature pyramid (FP) structures that applied SR-DAM to both low- and high-level feature maps. The first row indicates the same baseline method as in Table 5, which uses only ${{\textbf {F}}}_{5}$ without SR-DAM. The second row (Proposed w/o FP) indicates that SR-DAM was applied once to the high-level layers ${{\textbf {F}}}_{4}$ and ${{\textbf {F}}}_{5}$. The third row (Proposed) shows the proposed method in which SR-DAM is applied twice, once between ${{\textbf {F}}}_{4}$ and ${{\textbf {F}}}_{5}$, and again between ${{\textbf {F}}}_{3}$, ${{\textbf {F}}}_{4}$, and ${{\textbf {F}}}_{5}$. Because FP aggregates feature information at various levels, the proposed method that applied SR-DAM twice achieved the best performance, as shown in Table 6.

### Computation comparison

Table 7 shows a comparison of the number of parameters and the computation amounts of single and dual attention. PraNet, SANet and CBAM attention mechanisms use element-wise multiplication operations, and the channel-position attention used in DANet is based on self-attention. PraNet contains additional convolutional layers in parallel partial decoders and attention modules, which results in more computations than SANet.

Comparing CBAM and DANet, both models use ResNet-50 as the backbone and channel-position (spatial) attention modules. However, different variations in the computation were observed depending on the resolution of the input image owing to the difference between the use of channel-spatial attention in the two models. The proposed model exhibited an increased amount of computation compared to conventional dual attention-based methods (CBAM), but has the fewest parameters. CBAM exhibited poor performance compared to the proposed method, and DANet has many parameters. Therefore, the proposed method may be considered superior in terms of the balance of computation and parameters compared with the other methods.

## Conclusions

In this study, we proposed a model called SRaNet for polyp segmentation. The model can used to obtain various information about polyps using “SR-DAM” consisting of shallow attention and reverse attention mechanisms. Furthermore, we designed a softmax gate to effectively combine the two feature maps obtained from different types of attentions. The proposed SRaNet model was experimentally evaluated in comparison with a Res2Net baseline. To demonstrate the performance of our approach, we conducted comparative experiments not only with existing medical segmentation SOTA models but also with existing dual attention-based methods. In particular, the proposed model showed a large improvement in performance for unseen data, confirming that its generalization performance is better than that of the comparison methods. Furthermore, our model was more effective and explainable using intuitive and computationally efficient attention mechanisms. Our model leverages both shallow attention and reverse attention, specifically tailored to the characteristics of polyps. However, there are some limitations. We encountered segmentation errors with atypical images, such as colon images affected by glare. Also, this specialization might complicate feature extraction when the model is applied to different fields. In the future, we will focus on developing methods such as deblurring or multimodality module that demonstrate effective generalization capabilities, not only for polyp datasets but across various datasets as well. It is crucial to conduct large-scale clinical studies to transition bio-engineering technology from research to real-world clinical applications in hospitals. As a continuation of our work, we are taking steps to validate the clinical efficacy of our findings in a follow-up study.

## Data Availability

The datasets utilized in this research study, such as the Kvasir-SEG and the CVC-ClinicDB dataset can be individually found https://datasets.simula.no/kvasir-seg/ and https://polyp.grand-challenge.org/CVCClinicDB/, respectively. The overall training and test datasets can be found https://github.com/DengPingFan/PraNet.
